# HDGF Protects Retinal Pigment Epithelium from Glyoxal-Induced Ferroptosis via SIRT1/PGC-1α/Nrf2 Pathway

**DOI:** 10.3390/antiox14121434

**Published:** 2025-11-28

**Authors:** Heng-Dao Lin, Rong-Kung Tsai, Yao-Tseng Wen, Pei-Kang Liu

**Affiliations:** 1 Institute of Eye Research, Hualien Tzu Chi Hospital, Buddhist Tzu Chi Medical Foundation, Hualien 97403, Taiwan; 2 Institute of Medical Sciences, Tzu Chi University, Hualien 97403, Taiwan; 3 Doctoral Degree Program in Translational Medicine, Tzu Chi University and Academia Sinica, Hualien 97403, Taiwan; 4 Department of Ophthalmology, Kaohsiung Medical University Hospital, Kaohsiung Medical University, Kaohsiung 807377, Taiwan; 5 School of Medicine, College of Medicine, Kaohsiung Medical University, Kaohsiung 807378, Taiwan

**Keywords:** age-related macular degeneration (AMD), glyoxal (GO), hepatoma-derived growth factor (HDGF), mitochondria function, reactive oxygen species (ROS), ferroptosis

## Abstract

Age-related macular degeneration (AMD) is driven in part by the accumulation of reactive metabolites like glyoxal (GO), which induces retinal pigment epithelium (RPE) degeneration. Here, we demonstrate that GO triggers ferroptosis in human ARPE-19 cells, as characterized by iron-dependent lipid peroxidation, glutathione depletion, and reactive oxygen species (ROS) accumulation. This ferroptotic cell death is coupled with profound mitochondrial dysfunction, featuring network fragmentation and the downregulation of the key regulators MFN2, PGC-1α, and SIRT1. We identify hepatoma-derived growth factor (HDGF) as a potent protector against GO-induced damage. HDGF operates through a dual mechanism: it activates the p38 MAPK/AKT and SIRT1/PGC-1α axes to restore mitochondrial biogenesis and homeostasis, while concurrently enhancing the glutathione/GPX4 antioxidant system to suppress ferroptosis. This cytoprotective action is mediated via the PGC-1α/Nrf2 pathway, which integrates the enhancement of antioxidant defenses with the preservation of mitochondrial integrity. Our findings establish HDGF as a novel therapeutic agent for AMD, uniquely capable of concurrently targeting the interconnected pathways of ferroptosis and mitochondrial dysfunction, thereby addressing a critical unmet need in retinal disease treatment.

## 1. Introduction

Age-related macular degeneration (AMD) represents a growing global health challenge as a primary cause of irreversible vision loss in aging populations. While anti-VEGF therapies have transformed wet AMD management, the more prevalent dry AMD variant lacks effective treatments, creating an urgent need to elucidate its underlying molecular pathology [1]. Central to AMD progression is retinal pigment epithelium (RPE) dysfunction. RPE cells normally support photoreceptor survival through critical functions including metabolite exchange, retinoid recycling, and phagocytosis of photoreceptor outer segments [2]. Chronic oxidative stress mediated by reactive dicarbonyl compounds has been identified as a key driver of RPE degeneration, with glyoxal (GO), a stable byproduct of glucose metabolism and lipid oxidation, showing particular relevance in AMD pathogenesis [3,4]. Unlike transient reactive oxygen species, GO accumulates in RPE lipofuscin deposits and induces persistent oxidative damage that mirrors AMD’s chronic progression [5].

Recent advances have identified ferroptosis, an iron-catalyzed, lipid peroxidation-driven process, as contributing to AMD [6]. In the retina, this regulated cell death occurs in RPE cells exposed to oxidative stressors like sodium iodate and tert-butyl hydroperoxide (tBHP) [7,8], though its relationship with GO-induced damage remains unexplored. RPE susceptibility to ferroptosis may be exacerbated by age-related mitochondrial dysfunction, characterized by impaired fission–fusion dynamics that normally maintain mitochondrial homeostasis [9,10]. The delicate balance between mitochondrial fission and fusion, mediated by proteins such as Drp1, FIS1, mitofusins, and OPA1, becomes disrupted in aged RPE cells [9]. This dysfunction not only compromises cellular bioenergetics but also exacerbates oxidative stress through increased mitochondrial ROS production [11]. The resulting oxidative damage further depletes glutathione (GSH), the RPE’s primary antioxidant defense, creating a vicious cycle that may trigger either apoptosis [12,13] or ferroptosis [14] in RPE cells.

Hepatoma-derived growth factor (HDGF) has emerged as a promising neuro-protective agent through its ability to activate survival pathways in various neuronal systems [15,16]. In the retina, HDGF demonstrates protective effects on retinal ganglion cells through PI3K-AKT signaling [17], yet its potential role in preserving RPE viability against GO-induced damage has not been investigated. This study explores the novel hypothesis that HDGF protects RPE cells by simultaneously targeting GO-induced ferroptosis and mitochondrial dysfunction through coordinated activation of the SIRT1/PGC-1α/Nrf2 axis. Our findings provide new insights into AMD pathogenesis while identifying HDGF as a potential therapeutic candidate for this currently untreatable condition.

## 2. Materials and Methods

### 2.1. Cell Cultures and Treatment

The human retinal pigment epithelium (RPE) cell line ARPE-19 was obtained from the American Type Culture Collection (CRL-2302, ATCC; Manassas, VA, USA). Cells were maintained in Dulbecco’s Modified Eagle’s Medium (DMEM) supplemented with 1% fetal bovine serum (FBS, Gibco; Grand Island, NY, USA) and 1% penicillin/streptomycin (PS, Gibco; Grand Island, NY, USA) in a humidified incubator at 37 °C with 5% CO_2._ To induce polarization, cells were subjected to a serum-reduction protocol: FBS concentration was sequentially decreased from 10% to 5% for one week, then to 1% for approximately 4 weeks. This process yielded polarized RPE cells exhibiting characteristic markers (RPE65 and CRALBP), transepithelial electrical resistance, intact monolayer structure (ZO-1 immunostaining), and melanin production. Before treatment, ARPE-19 cells were seeded into 96-well plates or 6 cm Petri dishes at a density of 5 × 10^4^ cells/mL and incubated overnight. Following overnight culture, the medium was replaced with fresh medium. For inhibitor studies, cells were pretreated with the apoptosis inhibitor z-VAD or ferroptosis inhibitors (deferoxamine mesylate, DFO; ferrostatin-1, Fer-1) 3 h prior to subsequent treatments. In HDGF intervention experiments, cells were pretreated with varying concentrations of HDGF (75, 150, and 300 ng/mL) (CYT-681; Prospec; Rehovot, Israel) for 3 h. Subsequently, the cells were exposed to 4 mM glyoxal (GO, Sigma; Saint Louis, MO, USA) for an additional 24 h. The experimental design for this study is shown in Figure S1.

### 2.2. Cell Viability Assay

Cell viability of ARPE-19 cells was assessed using the CCK-8 assay. Cells were treated with varying concentrations of HDGF in the presence or absence of GO for 24 h. After treatment, the culture medium was replaced, and 10 μL of CCK-8 assay reagent (TEN-CCK8; Biotools; Taiwan) was added to each well. The plates were then incubated for 2 h at 37 °C in a 5% CO_2_ incubator. Finally, the absorbance of each well was measured at 450 nm using a microplate reader (BioTek; Santa Clara, CA, USA).

### 2.3. Lactate Dehydrogenase (LDH) Assay

Cytotoxicity was assessed using the lactate dehydrogenase (LDH) assay. LDH activity was measured using the CyQUANT™ LDH Cytotoxicity Assay Kit (C20301; Thermo Fisher Scientific; Waltham, MA, USA). Briefly, a positive control for LDH release was established by adding 10 μL of 10X lysis buffer to ARPE-19 cell samples and incubating for 30 min. Subsequently, 50 μL of the sample medium was transferred to a new plate, and 50 μL of reaction buffer was added to each well. The plate was then incubated at room temperature, protected from light, for 30 min. Absorbance was measured at 490 nm using a microplate reader (BioTek). The percentage cytotoxicity was calculated according to the manufacturer’s protocol and converted to relative cell survival rate.

### 2.4. Mitochondrial Membrane Potential (MMP) Detection

MMP was measured using the MitoProbe™ JC-1 Assay Kit (M34152; Thermo Fisher Scientific; Waltham, MA, USA). The JC-1 dye was reconstituted in 230 μL of dimethyl sulfoxide (DMSO, Sigma; Saint Louis, MO, USA) before use. Cells were washed three times with phosphate-buffered saline (PBS) and then incubated with a 1:100 dilution of JC-1 dye for 20–30 min. After incubation, cells were washed three times with PBS, and slides were analyzed using the EVOS M7000 imaging system (Thermo Fisher Scientific; Bothell, WA, USA).

### 2.5. Apoptosis Assay (Annexin V and PI)

Apoptosis was assessed using Annexin V and propidium iodide (PI) staining with a commercial kit (V13245; Thermo Fisher Scientific; Carlsbad, CA, USA). Following GO treatment, cells were washed with cold PBS, and 100 µL of 1X annexin-binding buffer was added to each well. Subsequently, 5 µL of Alexa Fluor™ 488 Annexin V and 1 µL of PI stock solution (100 µg/mL) were added to each well. Cells were then incubated for 15 min at room temperature (protected from light), washed twice with cold PBS, and analyzed using the EVOS M7000 imaging system (Thermo Fisher Scientific).

### 2.6. Measurement of Intracellular ROS Level (DCF-DA) and Mitochondrial Superoxide (MitoSOX Red)

Intracellular ROS generation was detected in living cells using the DCF-DA kit (R253; Dojindo; Japan), while mitochondrial superoxide levels were measured using the fluorogenic dye MitoSOX™ Red (M36008; Invitrogen; Waltham, MA, USA). Cells were seeded in 8-well chamber slides (PEZGS0816; Millipore; Burlington, MA, USA) at a density of 5 × 10^4^ cells/mL and incubated for 24 h at 37 °C. After incubation, cells were washed three times with PBS and treated with either 10 µM CM-H_2_DCFDA for 20 min or 5 µM Mito-SOX™ Red for 15 min in the dark. Following staining, cells were gently washed three times with PBS and counterstained with 1 µg/mL Hoechst 33342 (H21492; Invitrogen; Carlsbad, CA, USA) to visualize nuclei. Fluorescence images were captured and converted to binary images for quantification of average fluorescence intensity using ImageJ software v1.54g (NIH; Bethesda, MD, USA). Relative fluorescence units were normalized to the control group (set to 1). All experiments were performed in triplicate.

### 2.7. Intracellular Iron Measurement (FerroOrange)

Intracellular reactive iron (Fe^2+^) levels were measured in living cells using the FerroOrange kit (F374; Dojindo; Japan). Briefly, after 24 h of GO treatment, cells were washed three times with PBS. Subsequently, 1 µg/L of FerroOrange was added to the cells and incubated for 30 min at 37 °C. Stained cells were then imaged using the EVOS M7000 imaging system (Thermo Fisher Scientific).

### 2.8. Lipid Peroxidation Assay (BODIPY 581/591 C11)

The induction of lipid peroxidation was evaluated using the fluorescent lipid peroxidation sensor BODIPY 581/591 C11 (Thermo Fisher Scientific; Waltham, MA, USA), a lipophilic dye that exhibits a shift in fluorescence emission upon oxidation in cellular membranes. ARPE-19 cells were seeded in 8-well chamber slide and allowed to adhere for 24 h. Following adhesion, cells were loaded with 2 µM BODIPY 581/591 C11 in PBS for 30 min at 37 °C. After dye loading, cells were treated with GO for 24 h. Stained cells were then imaged using the EVOS M7000 imaging system (Thermo Fisher Scientific). The oxidized form of BODIPY was detected at an excitation/emission of 495/521 nm (green signal), while the non-oxidized form was measured at 575/600 nm (red signal). Lipid peroxidation was quantified as the ratio of oxidized (green) fluorescence to total fluorescence (oxidized + non-oxidized).

### 2.9. RNA Extraction and Quantitative Polymerase Chain Reaction (qPCR)

Briefly, 5 × 10^5^ ARPE-19 cells were seeded into 6 cm dishes and treated with GO for 24 h. Total RNA was isolated using the PureLink^®^ RNA Mini Kit (Thermo Fisher Scientific) following treatment. RNA was reverse-transcribed into cDNA using the iScript™ cDNA Synthesis Kit (Bio-Rad; Hercules, CA, USA). Quantitative PCR (qPCR) was performed using the QuantStudio™ 3 Real-Time PCR System (Thermo Fisher Scientific; San Jose, CA, USA). Gene expression levels were analyzed using the 2^−ΔΔCt^ method, with ACTB (β-actin) as the endogenous control. Target genes and corresponding primer sequences are listed in Table S1.

### 2.10. Western Blot

Protein samples (20–30 μg) were separated by electrophoresis using NuPAGE™ 4–12% Bis-Tris gels (NP0321BOX; Thermo Fisher Scientific) and transferred to PVDF membranes using iBlot™ 2 Transfer Stacks (IB24001; Invitrogen; Carlsbad, CA, USA). Membranes were blocked for 1 h at room temperature with Tris-buffered saline (TBS) containing 2% bovine serum albumin (BSA) and 0.05% Tween 20. After three 5 min washes with TBST (TBS with 0.05% Tween 20), membranes were incubated with primary antibodies diluted in blocking buffer at 4 °C overnight. Following three additional TBST washes, membranes were incubated with horseradish peroxidase (HRP)-conjugated secondary antibodies (anti-mouse or anti-rabbit) for 45 min at room temperature. After three final washes, protein bands were detected using chemiluminescent HRP substrate (Millipore; Temecula, CA, USA) and visualized with the iBright™ CL1500 imaging system (Thermo Fisher Scientific; Bothell, WA, USA). Protein concentrations were determined using the BCA assay. Band intensity was quantified using ImageJ software (NIH) with β-actin serving as the loading control for normalization. Three independent biological replicates were performed for all Western Blot analyses, and statistical significance was assessed using one-way ANOVA. All antibodies used are listed in Table S2.

### 2.11. Immunofluorescence Staining

ARPE-19 cells were seeded into 8-well chamber slides and incubated overnight. Cells were pretreated with or without HDGF (75, 150, or 300 ng/mL) for 3 h prior to 24-h exposure to 4 mM GO. Following treatment, cells were fixed with 4% paraformaldehyde for 15 min at room temperature and permeabilized with 0.1% Triton X-100 in PBS for 10 min. After three PBS washes, nonspecific binding was blocked with 2% BSA in PBS for 1 h at room temperature. Primary antibodies (listed in Table S2) diluted in 2% BSA/PBS were applied and incubated overnight at 4 °C. Cells were then incubated with fluorophore-conjugated secondary antibodies (FITC; 488 nm or 555 nm) in 2% BSA/PBS for 45 min at room temperature protected from light. Nuclei were counterstained with 1 μg/mL DAPI for 5 min, followed by three PBS washes. Fluorescence images were acquired using an EVOS M7000 imaging system (Thermo Fisher Scientific). Immunofluorescence intensity was quantified using ImageJ software (NIH), with values normalized to untreated controls.

### 2.12. Total and Reduced Glutathione Assay

Total glutathione (GSH + GSSG) and reduced glutathione (GSH) levels were measured using the Total Glutathione Colorimetric Assay Kit (ab239709; Abcam; Waltham, MA, USA). Following treatment, cells were washed three times with PBS and harvested in the provided lysis buffer. Cell lysates were centrifuged at 12,000× *g* for 10 min at 4 °C. For reduced GSH quantification, glutathione reductase was omitted from the reaction mixture. Absorbance was measured at 412 nm using a microplate reader. Standard curves from the kit were used to calculate absolute concentrations of reduced glutathione (μg/mL) and total glutathione (ng/mL). Protein concentrations were determined using the Pierce™ BCA Protein Assay Kit (23227; Thermo Scientific; Rockford, IL, USA) in 96-well microplates with BSA standards. Glutathione levels were normalized to total protein content (expressed as μg GSH/mg protein or ng GSSG/mg protein) for final quantification.

### 2.13. Statistical Analysis

All data are presented as mean ± standard deviation (SD) from at least three independent experiments. Statistical analyses were performed using GraphPad Prism 9 (GraphPad Software). Multiple group comparisons were conducted using one-way ANOVA followed by appropriate post-hoc tests. Statistical significance was set at *p* < 0.05, with significance levels denoted as follows: * *p* < 0.05 and ** *p* < 0.01.

## 3. Results

### 3.1. Non-Apoptotic Cell Death Induced by GO Exposure

**Figure 1:**
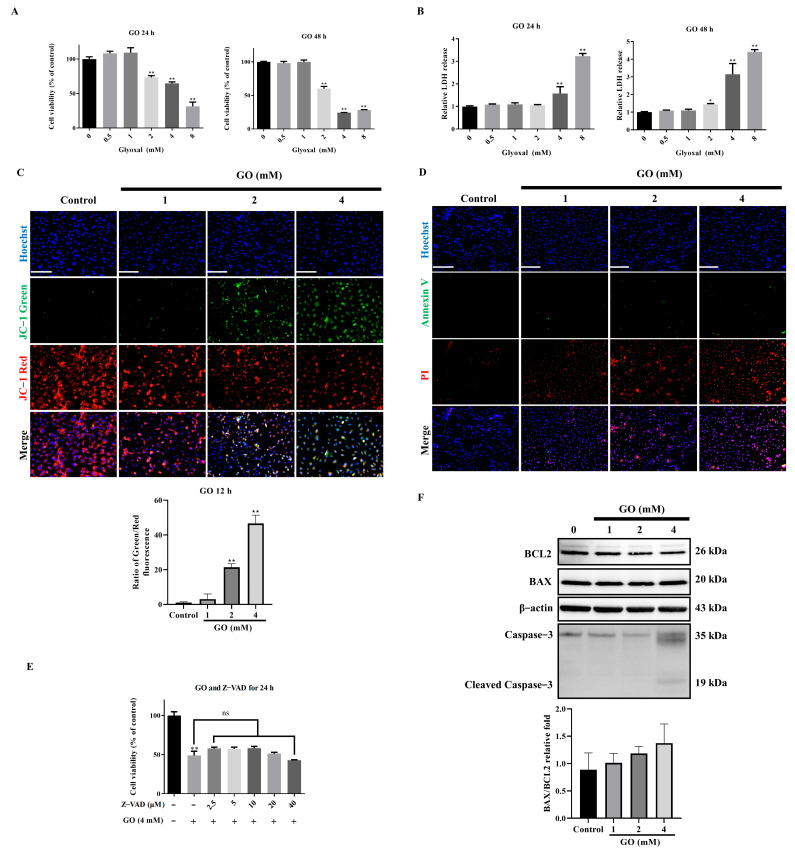
GO induces non-apoptotic cell death in ARPE-19 cells. (**A**) Cell viability assessed by CCK-8 assay after 24 h of GO treatment (0.5–8 mM) (*n* = 5). (**B**) LDH release measurement of membrane integrity (*n* = 5). (**C**) JC-1 staining showing MMP collapse (green: monomers; red: aggregates) (*n* = 3). (**D**) Annexin V/PI staining distinguishing apoptotic (AV^+^/PI^−^) and necrotic (PI^+^) populations (*n* = 3). (**E**) Cell viability with pan-caspase inhibitor Z-VAD pretreatment (*n* = 5). (**F**) Western Blot analysis of apoptosis markers (Bcl2, Bax, cleaved caspase-3) (*n* = 3). Data represent mean ± SD. Scale bars: 50 μm. * *p* < 0.05, ** *p* < 0.01 vs. control; ns: not significant.

Our investigation revealed that GO exposure induced ARPE-19 cell death through concentration- and time-dependent mechanisms. Cell viability assays demonstrated progressive cytotoxicity with increasing GO concentrations (Figure 1A), while LDH release measurements confirmed substantial plasma membrane disruption (Figure 1B), characteristic of necrotic cell death. Mitochondrial assessment using JC-1 staining showed a pronounced fluorescence shift from red to green (Figure 1C), indicating MMP collapse. Annexin V/PI staining revealed only a minor population of early apoptotic cells (Annexin V^+^/PI^−^), while the majority displayed late apoptotic/necrotic characteristics (PI^+^) (Figure 1D). The pan-caspase inhibitor Z-VAD failed to protect against GO-induced cytotoxicity (Figure 1E), and Western Blot analysis showed minimal changes in the Bax/Bcl2 ratio or caspase-3 cleavage (Figure 1F). These collective results demonstrate that GO-mediated ARPE-19 cell death occurs primarily through non-apoptotic pathways. The observed membrane integrity loss and cytoplasmic release strongly suggest necrosis-like mechanisms dominate the cell death process.

### 3.2. Ferroptosis Mediates GO-Induced RPE Cell Death

**Figure 2:**
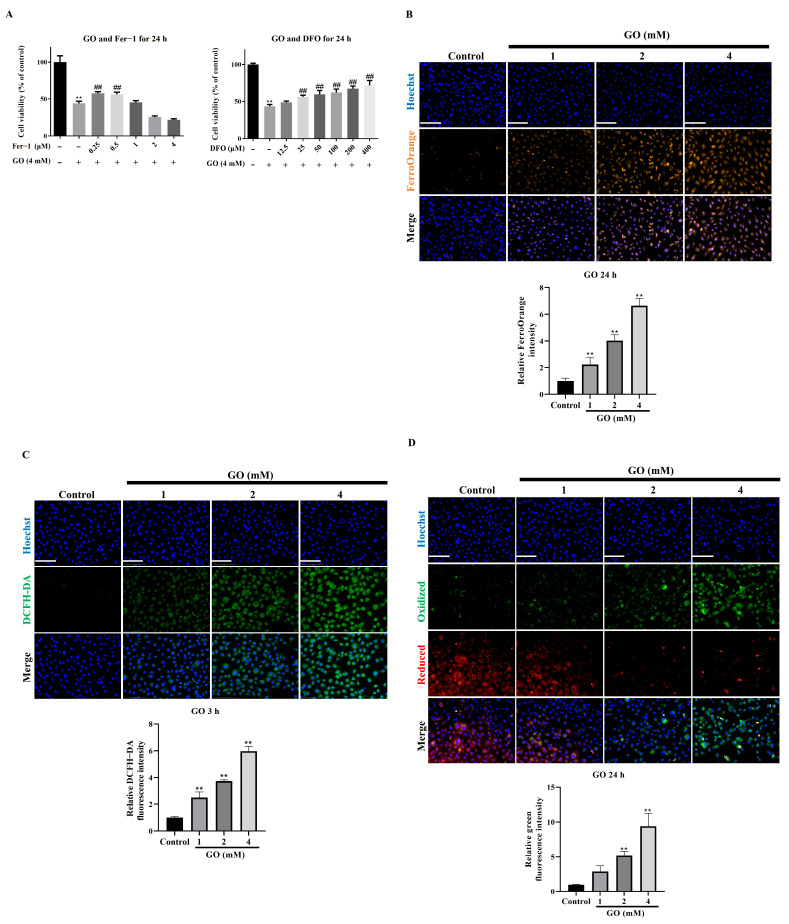
GO triggers ferroptosis in ARPE-19 cells. (**A**) Cell viability with ferroptosis inhibitor (DFO, Fer-1) (*n* = 5). (**B**) FerroOrange staining detecting Fe^2+^ accumulation (*n* = 3). (**C**) DCFDA fluorescence measuring intracellular ROS (*n* = 3). (**D**) BODIPY 581/591 C11 oxidation indicating lipid peroxidation (*n* = 3). Data represent mean ± SD. Scale bars: 50 μm. ** *p* < 0.01 vs. control and ^##^ *p* < 0.01 for GO vs. GO plus Fer-1 or DFO.

Ferroptosis, an iron and ROS dependent form of regulated cell death, has emerged as a critical mechanism in RPE cell degeneration [18]. Our investigation of GO-induced cytotoxicity revealed that the ferroptosis inhibitors ferrostatin-1 (Fer-1) and deferoxamine mesylate (DFO) significantly protected against GO-induced cell death (Figure 2A), strongly supporting ferroptosis involvement. Further evidence came from FerroOrange staining, which showed pronounced intracellular ferrous iron (Fe^2+^) accumulation in GO-treated cells through enhanced orange fluorescence (Figure 2B). In addition to iron, ferroptosis is driven by ROS generation and lipid peroxidation, processes initiated by the Fenton reaction [18,19]. The ferroptotic mechanism was further substantiated by measuring key downstream events. Using the fluorescent probe DCFH-DA, we observed GO concentration-dependent ROS elevation within 6 h of exposure (Figure 2C). Concurrently, BODIPY 581/591 C11 oxidation assays demonstrated markedly increased lipid peroxidation following 24 h of GO treatment (Figure 2D). These collective results establish that GO triggers ferroptosis as a primary cell death mechanism in ARPE-19 cells through iron accumulation, ROS generation, and lipid peroxidation.

### 3.3. GO Induces Oxidative Stress and Mitochondrial Impairment in RPE Cells

**Figure 3:**
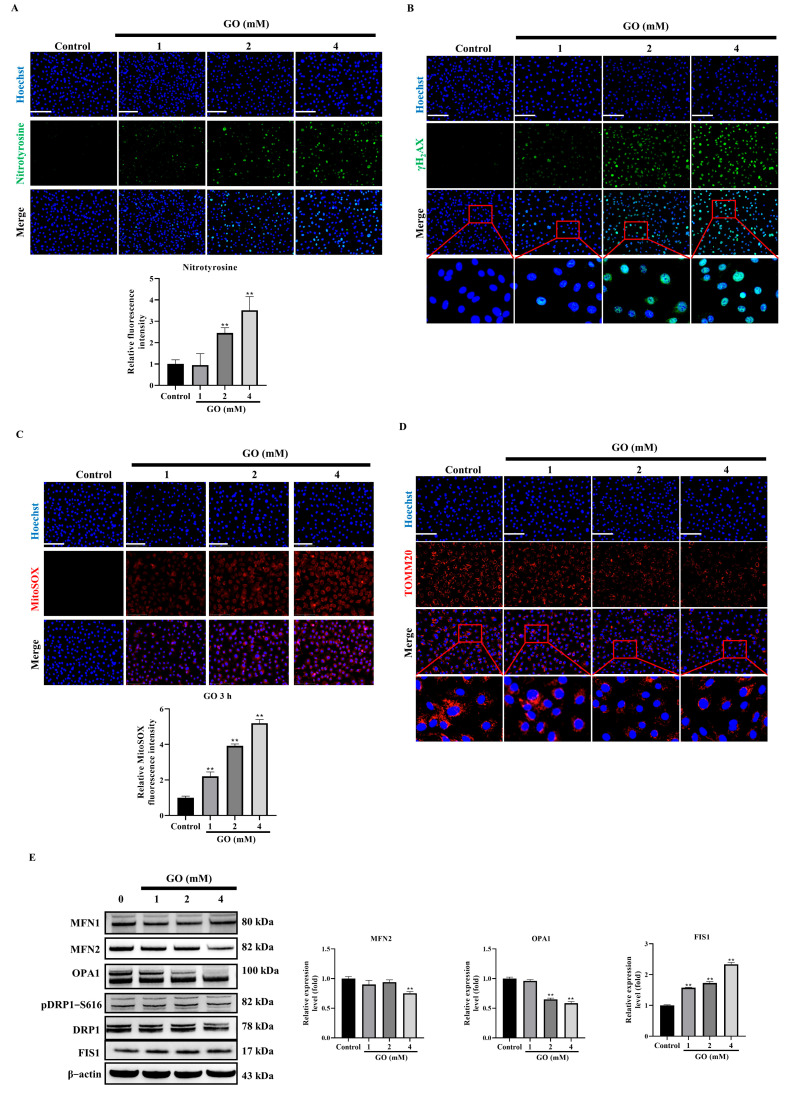
GO induces oxidative stress and mitochondrial dysfunction. (**A**) Nitrotyrosine immunofluorescence showing protein oxidation (*n* = 3). (**B**) γH_2_AX foci indicating DNA damage (*n* = 3). (**C**) MitoSOX Red detecting mitochondrial superoxide (*n* = 3). (**D**) Tomm20 staining revealing mitochondrial fragmentation (*n* = 3). (**E**) Western Blots of mitochondrial dynamics proteins (MFN1/2, FIS1, DRP1, p-DRP1 Ser616) (*n* = 3). Data represent mean ± SD. Scale bars: 50 μm. ** *p* < 0.01 vs. control.

Our investigation demonstrated that GO exposure induces extensive oxidative damage in ARPE 19 cells, as evidenced by significant increases in protein oxidation measured by 3-nitrotyrosine levels (Figure 3A) and DNA damage assessed through γH_2_AX foci (Figure 3B). Elevated oxidative stress in injured RPE cells often leads to mitochondrial dysfunction, compromising energy production and reducing MMP, ultimately contributing to RPE cell death. As indicated, mitochondrial superoxide levels significantly increased after 3 h of GO exposure (Figure 3C). Fluorescence microscopy revealed distinct alterations in mitochondrial morphology after GO treatment (Figure 3D). At the molecular level, GO treatment induced distinct changes in mitochondrial dynamics regulators. While DRP1 expression and phosphorylation status remained unchanged, we observed upregulation of the fission mediator FIS1. Conversely, mitochondrial fusion proteins showed marked suppression, such as mitofusin 2 (MFN2) and OPA1 (Figure 3E). These coordinated findings demonstrate that oxidative damage, mitochondrial network fragmentation, and altered expression of dynamics regulators collectively establish mitochondrial dysfunction as a central component of GO-induced RPE cell degeneration.

### 3.4. HDGF Protects RPE Cells Against GO-Induced Oxidative Damage

**Figure 4:**
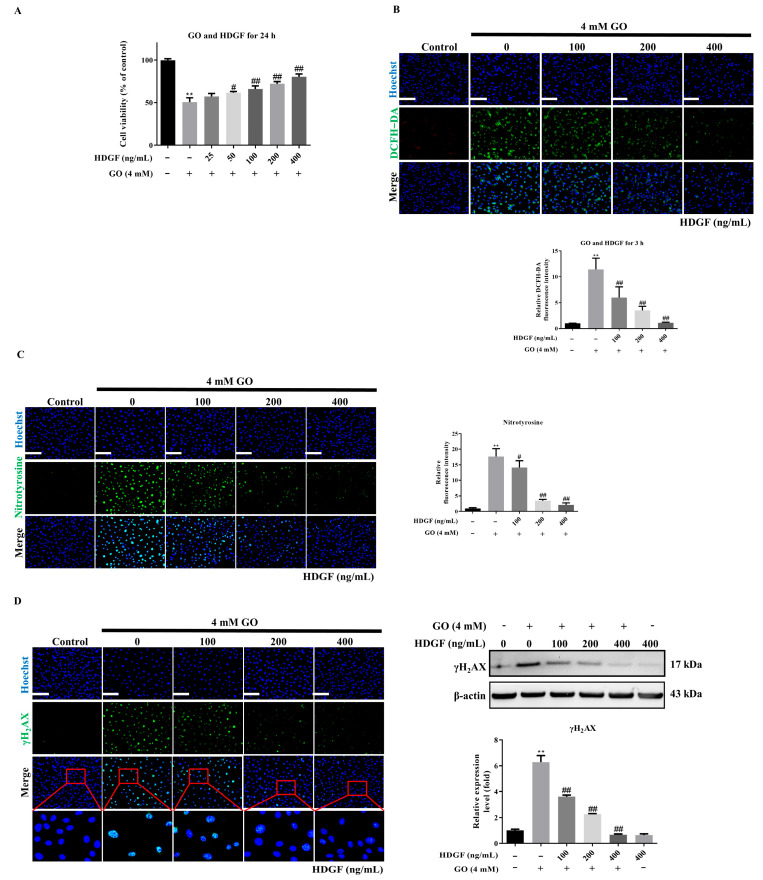
HDGF protects against GO-induced cytotoxicity. (**A**) HDGF (75–300 ng/mL) pretreatment preserves cell viability (*n* = 5). (**B**) DCFDA assay showing reduced ROS (*n* = 3). (**C**,**D**) Immunofluorescence of (**C**) 3-nitrotyrosine and (**D**) γH_2_AX demonstrating attenuated oxidative damage (*n* = 3). Data represent mean ± SD. Scale bars: 50 μm. ** *p* < 0.01 vs. control and ^#^ *p* < 0.05, ^##^ *p* < 0.01 for GO vs. GO plus HDGF.

A previous study has demonstrated the neuroprotective properties of HDGF in the retina [17]. Based on these findings, we investigated whether HDGF could mitigate GO-induced damage in ARPE 19 cells. Treatment with HDGF at concentrations of 75–300 ng/mL demonstrated a dose-dependent protective effect in GO treatment cells (Figure 4A), suggesting HDGF may inhibit oxidative stress generation. To evaluate this protective mechanism, we measured intracellular ROS levels along with markers of oxidative protein damage (3-nitrotyrosine) and DNA damage (γH_2_AX). HDGF treatment significantly decreased intracellular ROS levels compared to GO-treated cells (Figure 4B). Consistent with this, HDGF also attenuated GO-induced oxidative protein damage (Figure 4C) and DNA damage (Figure 4D) in ARPE-19 cells. These findings collectively suggest that HDGF protects ARPE-19 cells from GO-induced oxidative stress and cell death, highlighting its potential as a therapeutic agent for retinal degenerative conditions.

### 3.5. HDGF Regulates Nrf2 and Antioxidant Enzymes in GO-Treated RPE Cells

**Figure 5:**
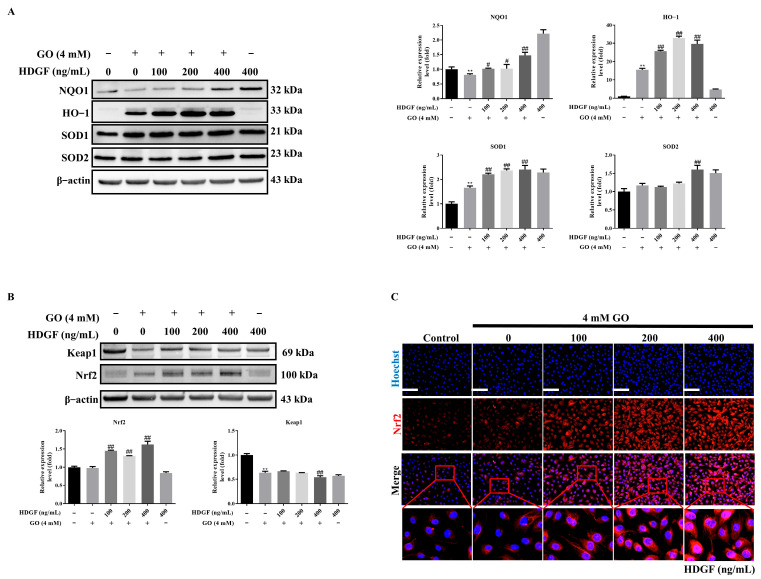
HDGF protects GO-induced ROS damage by induced anti-oxidative enzyme expression. (**A**) Western Blots of antioxidant enzymes (HO-1, NQO1, SOD1/2, catalase) (*n* = 3). (**B**) Nrf2 and Keap1 protein levels (*n* = 3). (**C**) Nrf2 nuclear translocation by immunofluorescence (*n* = 3). Data represent mean ± SD. Scale bars: 50 μm. ** *p* < 0.01 vs. control and ^#^ *p* < 0.05, ^##^ *p* < 0.01 for GO vs. GO plus HDGF.

Our investigation into the protective mechanisms of HDGF revealed its ability to potently activate the cellular antioxidant response system in GO-treated ARPE-19 cells. Our results demonstrated that HDGF significantly upregulated the expression of NQO1, HO-1, SOD1, and SOD2 compared to the GO-treated group (Figure 5A). This coordinated induction of phase II detoxification and antioxidant enzymes suggested potential Nrf2 pathway activation. Western Blot analysis confirmed that HDGF treatment increased total Nrf2 protein levels without altering Keap1 expression, indicating regulation through a Keap1-independent mechanism (Figure 5B). Immunofluorescence results provided visual evidence of Nrf2 pathway activation, demonstrating enhanced nuclear accumulation of Nrf2 in HDGF-treated cells (Figure 5C). These findings suggest that HDGF induces Nrf2 expression and activation, leading to the upregulation of antioxidant enzymes such as NQO1, HO-1, SOD1, and SOD2. This mechanism likely contributes to the alleviation of GO-induced oxidative stress in ARPE-19 cells.

### 3.6. HDGF Preserves Mitochondrial Function Through SIRT1/PGC-1α Signaling in GO-Treated RPE Cells

**Figure 6:**
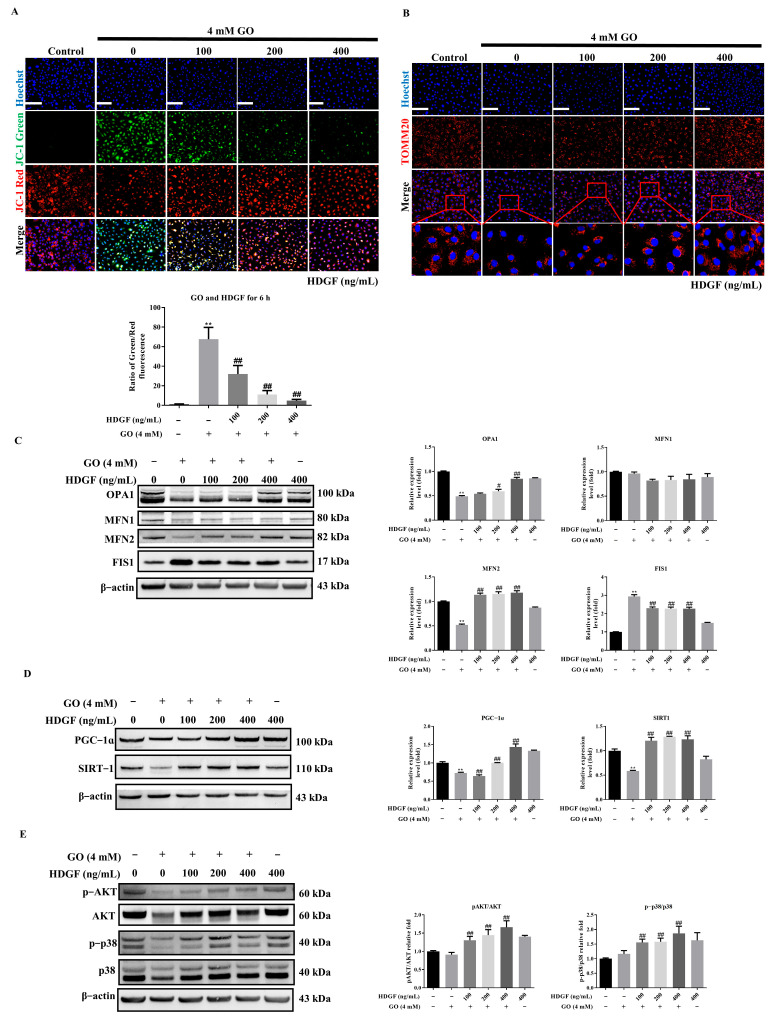
HDGF preserves mitochondrial function through SIRT1/PGC-1α. (**A**) JC-1 staining showing MMP preservation (*n* = 3). (**B**) Tomm20 staining of mitochondrial morphology (*n* = 3). (**C**) Western Blots of fusion (MFN1/2, OPA1) and fission (FIS1) proteins (*n* = 3). (**D**) SIRT1 and PGC-1α expression. (**E**) p38 MAPK and AKT phosphorylation status (*n* = 3). Data represent mean ± SD. Scale bars: 50 μm. ** *p* < 0.01 vs. control and ^#^ *p* < 0.05, ^##^ *p* < 0.01 for GO vs. GO plus HDGF.

To elucidate HDGF’s protective effects on mitochondrial function, we evaluated MMP and morphology. HDGF pretreatment significantly prevented GO-induced MMP loss (Figure 6A) and reduced mitochondrial fragmentation (Figure 6B). At the molecular level, HDGF treatment rebalanced mitochondrial dynamics by increasing the expression of fusion proteins OPA1 and MFN2 while reducing the fission mediator FIS1 (Figure 6C). These changes correlated with the rescue of key regulators of mitochondrial biogenesis, as HDGF restored SIRT1 expression and PGC-1α, both of which were significantly suppressed by GO treatment alone (Figure 6D). Since PGC-1α activity is modulated by AMPK, p38 MAPK, and AKT phosphorylation [20,21]. Our investigations revealed that HDGF treatment significantly enhanced the phosphorylation of both p38 MAPK and AKT, two key kinases that regulate PGC-1α transcriptional activity through post-translational modifications (Figure 6E). These findings demonstrate that HDGF’s mitochondrial protective effects are mediated through a multi-tiered signaling network involving p38 MAPK and AKT. The simultaneous activation of these pathways creates a synergistic effect that promotes mitochondrial biogenesis and enhances cellular resistance to GO-induced metabolic stress, offering potential therapeutic targets for retinal degenerative conditions associated with mitochondrial dysfunction.

### 3.7. HDGF Against GO-Induced Ferroptosis in ARPE-19 Cells

**Figure 7:**
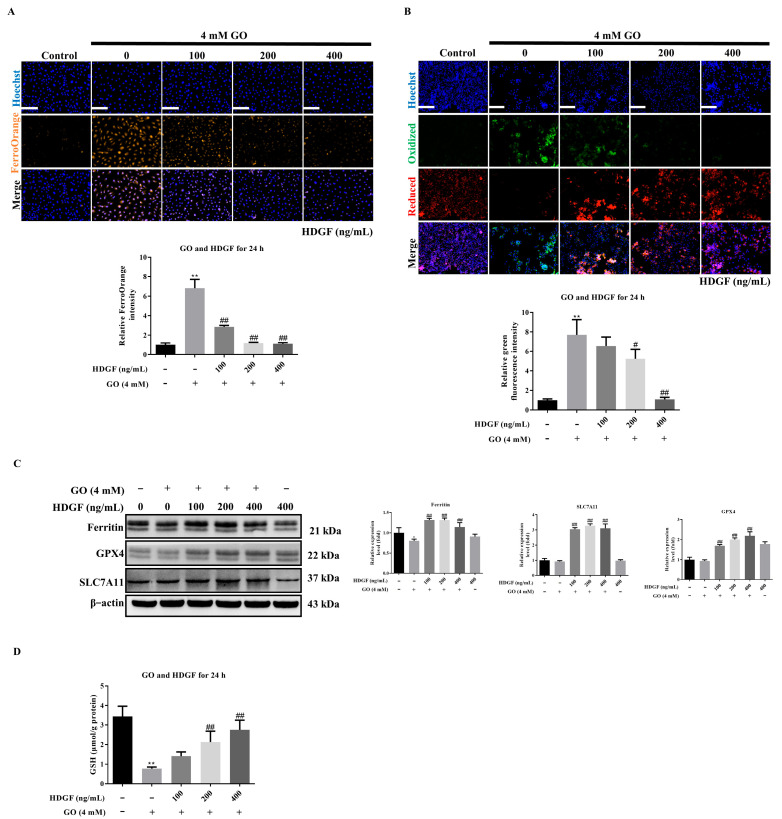
HDGF inhibits GO-induced ferroptosis. (**A**) FerroOrange staining showing reduced Fe^2+^ (*n* = 3). (**B**) BODIPY assay demonstrating decreased lipid peroxidation (*n* = 3). (**C**) Western Blots of GPX4, SLC7A11, and ferritin (*n* = 3). (**D**) GSH/GSSG ratio measurement (*n* = 3). Data represent mean ± SD from three independent experiments. Scale bars: 50 μm. * *p* < 0.05, ** *p* < 0.01 vs. control and ^#^ *p* < 0.05, ^##^ *p* < 0.01 for GO vs. GO plus HDGF.

Our investigation revealed that HDGF exerts comprehensive protection against ferroptosis in GO-treated ARPE-19 cells by targeting multiple nodes of the ferroptotic cascade. FerroOrange staining demonstrated that HDGF pretreatment reduced intracellular ferrous iron accumulation compared to GO-treated controls (Figure 7A). This reduction in redox-active iron correlated with a significant decrease in lipid peroxidation by decreased fluorescence intensity of BODIPY 581/591 C11 in HDGF-treated cells (Figure 7B). To elucidate the molecular mechanisms underlying HDGF’s protective effects. Western Blotting revealed that HDGF restored ferritin expression compared to the GO-treated group, reversing GO-induced suppression of this critical iron storage protein. Furthermore, HDGF treatment upregulated two key components of the glutathione antioxidant system, increasing GPX4 and SLC7A11 expression compared to GO-treated cells (Figure 7C). These molecular changes translated to functional improvements in cellular redox status, with HDGF pretreatment restoring intracellular GSH levels (Figure 7D). The coordinated effects of HDGF on iron regulation, lipid peroxidation inhibition, and glutathione system enhancement demonstrate its multi-targeted protection against ferroptosis. By simultaneously addressing multiple points of vulnerability in the ferroptotic cascade, HDGF provides robust defense against GO-induced oxidative damage in RPE cells.

## 4. Discussion

Our study provides compelling evidence that GO induces ferroptosis in RPE cells through iron-dependent lipid peroxidation, establishing a novel mechanism of RPE degeneration relevant to AMD pathogenesis. These findings demonstrate that while GO exposure causes mitochondrial dysfunction and oxidative stress, classical apoptosis does not represent the primary cell death pathway. Instead, ferroptosis emerges as the dominant mechanism, as evidenced by the protective effects of specific inhibitors (DFO and Fer-1) and characteristic biochemical markers including iron accumulation, glutathione depletion, and lipid peroxidation. The protective effect of HDGF presents a compelling therapeutic profile for dry AMD. While HDGF exhibits pro-angiogenic properties in other contexts, its application in dry AMD is supported by several key considerations. The intact blood-retinal barrier in this condition likely mitigates neovascularization risks, while HDGF’s demonstrated cytoprotection against oxidative stress directly addresses the core pathology. Existing retinal studies showing no adverse fibrovascular effects further support HDGF’s potential as a targeted strategy for dry AMD, where preserving RPE function is paramount. Growing evidence suggests that ferroptosis may represent a final common pathway in various forms of retinal degeneration, characterized by the triad of iron accumulation, lipid peroxidation, and glutathione depletion [14,22]. These pathological changes mirror observations in AMD donor eyes, where elevated iron levels and oxidative damage markers have been consistently reported [23,24]. The particular vulnerability of RPE to ferroptosis likely stems from its high metabolic activity, constant light exposure, and substantial polyunsaturated fatty acid content in outer segment membranes [6]. Compared with commonly used hydrogen peroxide (H_2_O_2_) and tert-butyl hydroperoxide (tBHP, a stable form of H_2_O_2_), the glyoxal-treated model better mimics the age-related oxidative stress patterns associated with intracellular accumulation of advanced glycation end-products (AGEs) in retinal diseases [5].

The mitochondrial fragmentation and bioenergetic failure we observed in GO-treated RPE cells recapitulate key features of AMD pathology [25,26]. Our results reveal that GO selectively disrupts mitochondrial dynamics by suppressing fusion proteins (MFN2 and OPA1) while upregulating the fission mediator FIS1, without affecting DRP1 activation. This pattern differs from canonical mitochondrial fragmentation pathways and suggests GO may trigger a unique form of mitochondrial dysfunction. Interestingly, we don’t observe significant inhibition of mitophagy marker mRNA levels such as *cathepsin D*, *LAMP2*, *ATG7*, *BNI3L*, *COX4l1*, *PINK1*, and FundC1 in GO-treated cells (Figure S2A). In contrast, we found GO can induce some autophagy and lysosome mRNA markers such as *MAP1LC3B*, *ATG4D*, *LAM1*, *ATG5*, and *cathepsin B* (Figure S2B). In addition, we found GO-induced autophagy (p62 degradation and LC3 induction) and cathepsin D, which is reversed by HDGF treatment (Figure S3). These results suggest that GO doesn’t inhibit mitophagy and may instead induce autophagy, leading to cell growth inhibition or death, as Chang et al. (2015) described [27].

In a previous study, HDGF exhibited robust survival-promoting effects on injured adult RGCs via activation of the PI3K-AKT pathway, which has powerful clinical application value [17]. Extending these findings, our current study reveals that HDGF also effectively counteracts GO-induced RPE degeneration through multiple complementary mechanisms. While our comparative analysis of different oxidative stressors (NaIO_3_, GO, and MGO) showed that HDGF did not significantly enhance Nrf2 and HO-1 mRNA expression levels (Figure S4), we observed a notable upregulation of their corresponding proteins. This discrepancy between transcriptional and translational responses suggests that HDGF may regulate the Nrf2-HO-1 pathway primarily through post-transcriptional mechanisms, potentially involving enhanced protein stability or translational efficiency. This post-transcriptional enhancement of key antioxidant proteins, along with the upregulation of other protective enzymes (NQO1, SOD1/2), provides crucial protection against oxidative stress and ferroptotic cell death. HDGF demonstrated remarkable efficacy in counteracting GO-induced RPE degeneration through multiple complementary mechanisms in this study. The upregulation of Nrf2 and its downstream antioxidant enzymes (NQO1, HO-1, SOD1/2) provided additional protection against oxidative stress and ferroptotic cell death. Notably, HDGF’s effects on Nrf2 activation occurred independently of Keap1 degradation, suggesting alternative regulatory mechanisms that warrant further investigation. In addition, HDGF treatment not only reduced oxidative stress markers but also restored mitochondrial homeostasis by rebalancing fission/fusion dynamics and activating the SIRT1/PGC-1α axis. Notably, the relationship between SIRT1 and PGC-1α within this protective pathway reveals a sophisticated regulatory mechanism. SIRT1 functions as a crucial metabolic sensor that responds to GO-induced stress conditions, where it deacetylates and activates PGC-1α, establishing a positive feedback loop that amplifies the cellular defense response. Our findings demonstrate that HDGF-mediated activation of the SIRT1/PGC-1α pathway represents a crucial protective mechanism against GO-induced RPE degeneration. SIRT1, an NAD^+^-dependent deacetylase, plays a pivotal role in retinal protection by coordinating mitochondrial biogenesis through PGC-1α activation [28,29] and enhancing antioxidant defense via Nrf2 deacetylation [30]. In the context of AMD pathology, this regulatory axis assumes particular significance. The age-related decline in SIRT1 activity, exacerbated by chronic oxidative stress from GO exposure, creates a vulnerable environment in RPE cells that mirrors dry AMD progression. HDGF intervention effectively restores this compromised pathway, thereby enhancing mitochondrial quality control and cellular resilience. This SIRT1/PGC-1α/Nrf2 signaling cascade provides a mechanistic framework through which HDGF exerts its cytoprotective effects, ultimately leading to improved mitochondrial function and enhanced oxidative stress resistance. These coordinated pathways are particularly critical in RPE cells, which must sustain robust mitochondrial function while continuously counteracting oxidative insults from light exposure and high metabolic demand. The age-related decline in SIRT1 activity contributes to AMD progression by impairing mitochondrial homeostasis and exacerbating oxidative stress, as supported by genetic and biochemical evidence linking SIRT1 polymorphisms to AMD risk [31]. Our findings not only align with but also extend previous research on SIRT1’s cytoprotective mechanisms in retinal cells [32]. Importantly, we further demonstrate that HDGF exerts complementary protective effects by regulating PGC-1α through the p38 MAPK and AKT signaling pathways. This multi-level regulation—through both SIRT1-mediated activation and kinase-mediated stabilization—ensures sustained PGC-1α activity and comprehensive protection against persistent oxidative challenges in the RPE microenvironment. The phosphorylation of PGC-1α by p38 MAPK enhances PGC-1α stability and transcriptional activity, providing crucial regulation of this short-lived (2–3 h half-life) metabolic master regulator [33,34]. Furthermore, p38 MAPK-mediated phosphorylation disrupts the inhibitory interaction between PGC-1α and the p160MBP corepressor in myoblasts, thereby significantly increasing PGC-1α’s transactivation capacity and promoting its downstream effects on mitochondrial biogenesis and cellular metabolism [35]. Similarly, AKT regulates PGC-1α by phosphorylation-dependent stabilization, nuclear retention, and enhanced transcriptional activity [36,37,38], synergizing with SIRT1/p38 MAPK to sustain mitochondrial function. Recent studies have reported that GPX4 deficiency in RPE induces chronic oxidative stress and degeneration resembling AMD, highlighting the critical role of ferroptosis in retinal pathology [39,40]. Our findings demonstrate that GO-induced ferroptosis in RPE cells occurs through a cascade initiated by thiol group oxidation, leading to GSH depletion, labile iron release, and subsequent ROS generation. This process is tightly regulated by system Xc^−^, the cystine/glutamate antiporter composed of SLC7A11 and SLC3A2, which maintains intracellular cysteine levels essential for GSH synthesis [41]. Notably, we identified HDGF as a potent inhibitor of GO-induced ferroptosis through multiple protective mechanisms. HDGF not only preserves GPX4 activity, preventing lipid peroxide accumulation but also upregulates SLC7A11 expression to enhance cystine uptake and GSH biosynthesis. Furthermore, HDGF restores ferritin expression, suggesting that its ability to stabilize iron homeostasis and limit the availability of redox-active iron drives ferroptosis. These coordinated actions of HDGF on both the antioxidant and iron regulatory systems provide comprehensive protection against ferroptosis.

## 5. Conclusions

In conclusion, our study elucidates a comprehensive protective mechanism by which HDGF counters GO-induced ferroptosis in RPE cells through integrated activation of pAKT/p-p38 signaling and the SIRT1/PGC-1α axis. These coordinated pathways collectively mitigate oxidative stress by restoring glutathione homeostasis, reducing iron-mediated lipid peroxidation, and preserving mitochondrial function through enhanced biogenesis and dynamics. The multifaceted action of HDGF addresses core pathological features of AMD, including chronic oxidative damage and metabolic dysfunction in RPE cells. These findings not only advance our mechanistic understanding of RPE degeneration but also identify HDGF as a promising therapeutic candidate for AMD. Its ability to simultaneously target ferroptosis pathways and mitochondrial quality control systems provides a unique advantage over single-target approaches. Future studies should prioritize preclinical validation in animal models of retinal degeneration to assess HDGF’s efficacy and safety profile before clinical translation.

## Data Availability

The original contributions presented in this study are included in the article/Supplementary Materials. Further inquiries can be directed to the corresponding author.

## Supplementary Materials

The following supporting information can be downloaded at: https://www.mdpi.com/article/10.3390/antiox14121434/s1. Figure S1: Schematic diagram of the experimental design. Timeline of experiment, including cell culture and treatment. (A) ARPE-19 cells were pretreated with or without inhibitors (Fer-1, DFO or Z-VAD) for 3 h before GO (4 mM) exposure for 24 or 48 h to assess cell death mechanisms. (B) For protection assays, cells were pretreated with HDGF (75–300 ng/mL) for 3 h before GO (4 mM) challenge for 24 h to evaluate therapeutic effects. All experiments included matched controls and were repeated in three independent biological replicates; Figure S2: qPCR analysis of autophagy and mitophagy-related genes. ARPE-19 cells were treated with 2 mM GO for 24 h and examined mRNA expression level by qPCR. (A) Mitophagy-related genes such as *cathepsin D*, *LAMP2*, *ATG7*, *BNIP3L*, *COX4l1*, *PINK1* and *FundC1* were exam by qPCR (*n* = 3). (B) Autophagy-related gene such as *MAP1LC3B*, *ATG4D*, *LAM1*, *ATG5*, *cathepsin B* (*n* = 3). Data represent mean ± SD. Values are statistically significant at ** *p* < 0.01 vs. control; Figure S3: HDGF attenuates GO-induced autophagy in ARPE-19 cells. ARPE-19 cells were treated with HDGF (75–300 ng/mL) for 3 h and following treated with 4 mM GO for 24 h. The protein expression level of cathepsin D, p62 and LC3 has been assessed by Western Blot (*n* = 3). Data represent mean ± SD. (** *p* < 0.01 vs. control and ^#^ *p* < 0.05, ^##^ *p* < 0.01 for GO vs. GO plus HDGF). Figure S4: qPCR analysis of Nrf2 and HO-1 expression level. ARPE-19 cells were treated with NaIO_3_ (6 mM), GO (2 mM) or MGO (1 mM) with or without HDGF for 24 h and examined Nrf2 and HO-1 mRNA expression level by qPCR (*n* = 3). Data represent mean ± SD. (** *p* < 0.01 vs. control and, ^##^ *p* < 0.01 for Oxidative stress vs. for Oxidative stress plus HDGF); Table S1: List of qPCR primer sequences used in this study; Table S2: List of primary and secondary antibodies used for Western Blot (WB) and Immunofluorescence (IF).

## Abbreviations

The following abbreviations are used in this manuscript:

AMD	Age-related macular degeneration
RPE	Retinal pigment epithelium
GO	Glyoxal
tBHP	Tert-butyl hydroperoxide
GSH	Glutathione
HDGF	Hepatoma-derived growth factor
DMEM	Dulbecco’s Modified Eagle’s Medium
PS	Penicillin/Streptomycin
FBS	Fetal bovine serum
LDH	Lactate dehydrogenase
MMP	Mitochondrial membrane potential
PBS	Phosphate-buffered saline
DMSO	Dimethyl sulfoxide
PI	Propidium iodide
qPCR	Quantitative polymerase chain reaction
TBS	Tris-buffered saline
HRP	Horseradish peroxidase
DFO	Deferoxamine mesylate
Fer-1	Ferrostatin-1
Fe^2+^	Ferrous iron
MFN2	Mitofusin 2
H_2_O_2_	Hydrogen peroxide
AGEs	Advanced glycation end-products
WB	Western Blot
IF	Immunofluorescence
