# Supplementary material for: HDGF Protects Retinal Pigment Epithelium from Glyoxal-Induced Ferroptosis via SIRT1/PGC-1α/Nrf2 Pathway
Source: Antioxidants (Basel). 2025 Nov 28;14(12):1434. doi: 10.3390/antiox14121434 (PMC12729632; doi:10.3390/antiox14121434)
Supplement: Supplementary file 1 [file antioxidants-14-01434-s001.zip › antioxidants-3946914-Table S1.pdf]

Table S1. List of qPCR primer sequences used in this study

Gene name	Accession number	Forward (5' to 3')	Reverse (5' to 3')
MAP1LC3B	NM_022818.5	GAGAAGACCTTCAAGCAGCG	TATCACCGGGATTTTGGTTG
ATG4D	NM_032885.6	CTCAACCCCGTGTATGTGC	TACAGTGAGTGTCGCGGTTT
ATG5	NM_004849.4	AAAGATGTGCTTCGAGATGTGT	CACTTTGTCAGTTACCAACGTCA
cathepsin B	NM_147783.4	GAGCTGGTCAACTATGTCAACA	GCTCATGTCCACGTTGTAGAAGT
cathepsin D	NM_001909.5	TGCTCAAGAACTACATGGACGC	CGAAGACGACTGTGAAGCACT
LAMP2	NM_001122606.1	GAAAATGCCACTTGCCTTTATGC	AGGAAAAGCCAGGTCCGAAC
ATG7	NM_001144912.2	CAGTTTGCCCCTTTTAGTAGTGC	CCAGCCGATACTCGTTCAGC
BNIP3L	NM_004331.3	ATGTCGTCCCACCTAGTCGAG	TGAGGATGGTACGTGTTCCAG
PINK1	NM_032409	GCCTCATCGAGGAAAAACAGG	GTCTCGTGTCCAACGGGTC
FundC1	NM_173794	CCTCCCCAAGACTATGAAAGTGA	AAACACTCGATTCCACCACTG
ACTB	NM_001101.5	ACCGCGAGAAGATGACCCAG	GGATAGCACAGCCTGGATAGCAA
