# Supplementary material for: HDGF Protects Retinal Pigment Epithelium from Glyoxal-Induced Ferroptosis via SIRT1/PGC-1α/Nrf2 Pathway
Source: Antioxidants (Basel). 2025 Nov 28;14(12):1434. doi: 10.3390/antiox14121434 (PMC12729632; doi:10.3390/antiox14121434)
Supplement: Supplementary file 1 [file antioxidants-14-01434-s001.zip › antioxidants-3946914-Table S2.pdf]

Table 2. List of primary and secondary antibodies used for Western Blot (WB) and Immunofluorescence (IF)

Primary Antibodies	Company	Catalog number	Dilution
BCL2	Cell Signaling	ab194583	1:1000 (WB)
BAX	Cell Signaling	# 2772	1:1000 (WB)
β-actin	sigma	MAB1501	1:10000 (WB)
γH_2AX	Invitrogen	MA1-2022	1:1000 (WB)
MFN1	Cell Signaling	#14739	1:1000 (WB)
MFN2	Cell Signaling	#9482	1:1000 (WB)
OPA1	Cell Signaling	＃80471	1:1000 (WB)
pDRP1-S616	Cell Signaling	#3455	1:1000 (WB)
DRP1	Cell Signaling	#5391	1:1000 (WB)
FIS1	Invitrogen	PA5-22142	1:2000 (WB)
NQO1	Abcam	ab239709	1:1000 (WB)
HO1	Abcam	ab239709	1:3000 (WB)
SOD1	Abcam	ab13498	1:1000 (WB)
SOD2	Invitrogen	PA5-30604	1:1000 (WB)
Keap1	Abcam	ab119403	1:1000 (WB)
Nrf2	Abcam	ab137550	1:1000 (WB)
p-AKT	Cell Signaling	#4060	1:1000 (WB)
AKT	Cell Signaling	#4298	1:1000 (WB)
p-p38	Cell Signaling	#4060	1:1000 (WB)
p38	Cell Signaling	#33-1300	1:1000 (WB)
Ferritin	Abcam	ab75973	1:1000 (WB)
GPX4	Abcam	ab125066	1:1000 (WB)
SLC7A11	Abcam	ab307601	1:1000 (WB)
cathepsin D	Cell Signaling	#2284	1:1000 (WB)
p62	Abcam	ab109012	1:1000 (WB)
LC3	Invitrogen	PA1-16931	1:1000 (WB)
3-Nitrotyrosine	Abcam	ab110282	1:200 (IF)
γH_2AX	Invitrogen	MA1-2022	1:200 (IF)
TOMM20	Invitrogen	MA5-32148	1:200 (IF)
Nrf2	Abcam	ab137550	1:200 (IF)
**Secondary Antibodies**	**Company**	**Catalog number**	**Dilution**
Rabbit anti-Mouse IgG (H+L) Secondary Antibody, HRP	Invitrogen	61-6520	1:5000 (WB)
Goat anti-Mouse IgG (H+L) Secondary	Invitrogen	31430	1:5000 (WB)

Antibody, HRP			
488 goat anti-mouse IgG (H+L)	Invitrogen	A32723	1:400 (IF)
488 goat anti-rabbit IgG (H+L)	Invitrogen	A32732	1:400 (IF)
